# Supplementary material for: A high-resolution mRNA expression time course of embryonic development in zebrafish
Source: eLife. 2017 Nov 16;6:e30860. doi: 10.7554/eLife.30860 (PMC5690287; doi:10.7554/eLife.30860)
Supplement: Supplementary file 6. [file elife-30860-supp6.zip › biolayout-clusters-files/Cluster205-genes.html]

Cluster205


# Cluster205: Genes

| Ensembl ID | Gene Name | Chr | Start | End | Biotype |
| --- | --- | --- | --- | --- | --- |
| ENSDARG00000095142 | ENSDARG00000095142 | 5 | 32231325 | 32259545 | protein\_coding |
| ENSDARG00000079725 | dhx9 | 2 | 35871785 | 35891133 | protein\_coding |
| ENSDARG00000030340 | guk1a | 2 | 3186685 | 3203882 | protein\_coding |
| ENSDARG00000052641 | kpna3 | 1 | 45975464 | 46015407 | protein\_coding |
| ENSDARG00000042627 | nhsl1b | 20 | 30822700 | 30987041 | protein\_coding |
| ENSDARG00000074321 | tead3a | 23 | 5563321 | 5624393 | protein\_coding |
| ENSDARG00000015554 | zic2a | 9 | 31471415 | 31476264 | protein\_coding |
